# Supplementary material for: Influence of the Alcohols on the ZnO Synthesis and Its Properties: The Photocatalytic and Antimicrobial Activities
Source: Pharmaceutics. 2022 Dec 18;14(12):2842. doi: 10.3390/pharmaceutics14122842 (PMC9783502; doi:10.3390/pharmaceutics14122842)
Supplement: Supplementary file 1 [file pharmaceutics-14-02842-s001.zip › pharmaceutics-2048602-supplementary.pdf]

pharmaceutics

Influence of the alcohols on the ZnO synthesis and its properties. The photocatalytic and antimicrobial activities

Ludmila Motelica [1,2], Bogdan-Stefan Vasile [1,2,3], Anton Ficai [1,2,3,4], Adrian-Vasile Surdu [1,2,3], Denisa Ficai [1,2,3], Ovidiu–Cristian Oprea [1,2,3,4*], Ecaterina Andronescu [1,2,3,4], Dan Corneliu Jinga[5], Alina Maria Holban[6]

1 National Research Center for Micro and Nanomaterials, University Politehnica of Bucharest, 060042 Bucharest, Romania
2 National Research Center for Food Safety, University Politehnica of Bucharest, Splaiul Independentei 313, 060042 Bucharest, Romania
3 Faculty of Chemical Engineering and Biotechnologies, University Politehnica of Bucharest, 1-7 Polizu St., 011061 Bucharest, Romania;
4 Academy of Romanian Scientists, Ilfov Street 3, 050044 Bucharest, Romania
5 Department of Medical Oncology, Neolife Medical Center, Ficusului Bd. 40, 077190 Bucharest, Romania
6 Microbiology & Immunology Department, Faculty of Biology, University of Bucharest, 077206 Bucharest, Romania
* Correspondence: ovidiu.oprea@upb.ro or ovidiu73@yahoo.com

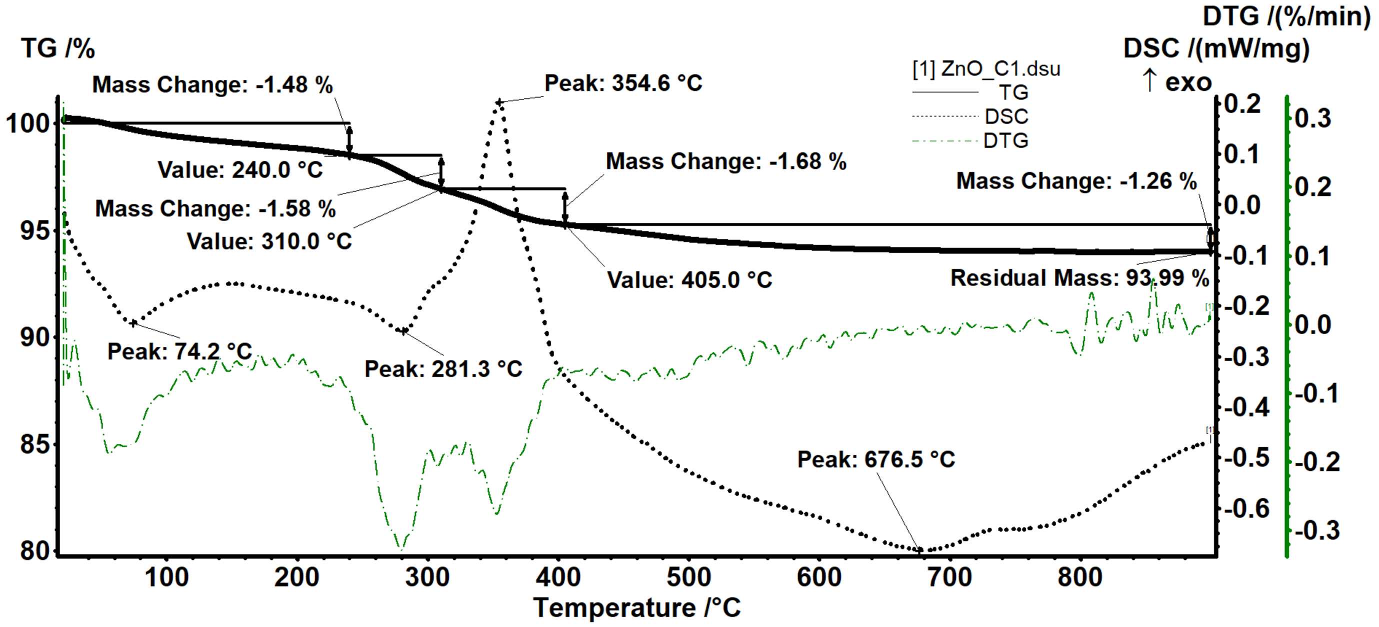

Figure S1. The thermal analysis, TG –DTG – DSC for the sample ZnO_C1 – the nanopowder obtained from zinc acetate by using methanol as solvent.

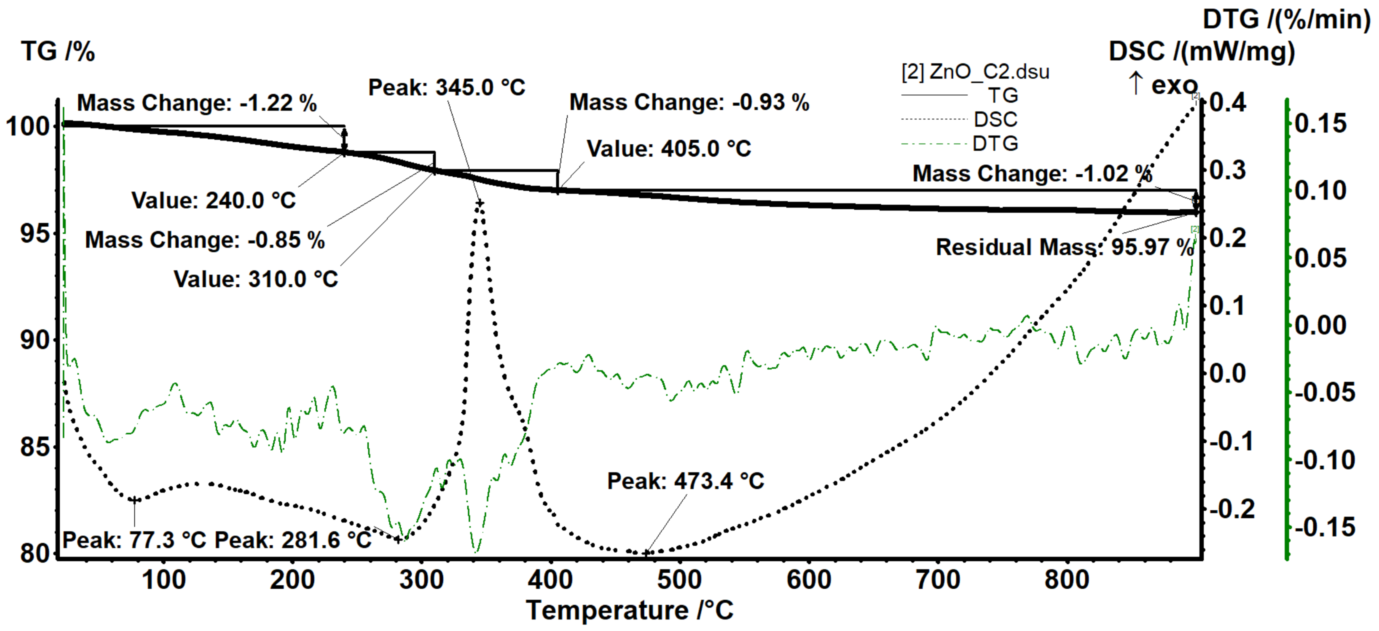

Figure S2. The thermal analysis, TG –DTG – DSC for the sample ZnO_C2 – the nanopowder obtained from zinc acetate by using ethanol as solvent.

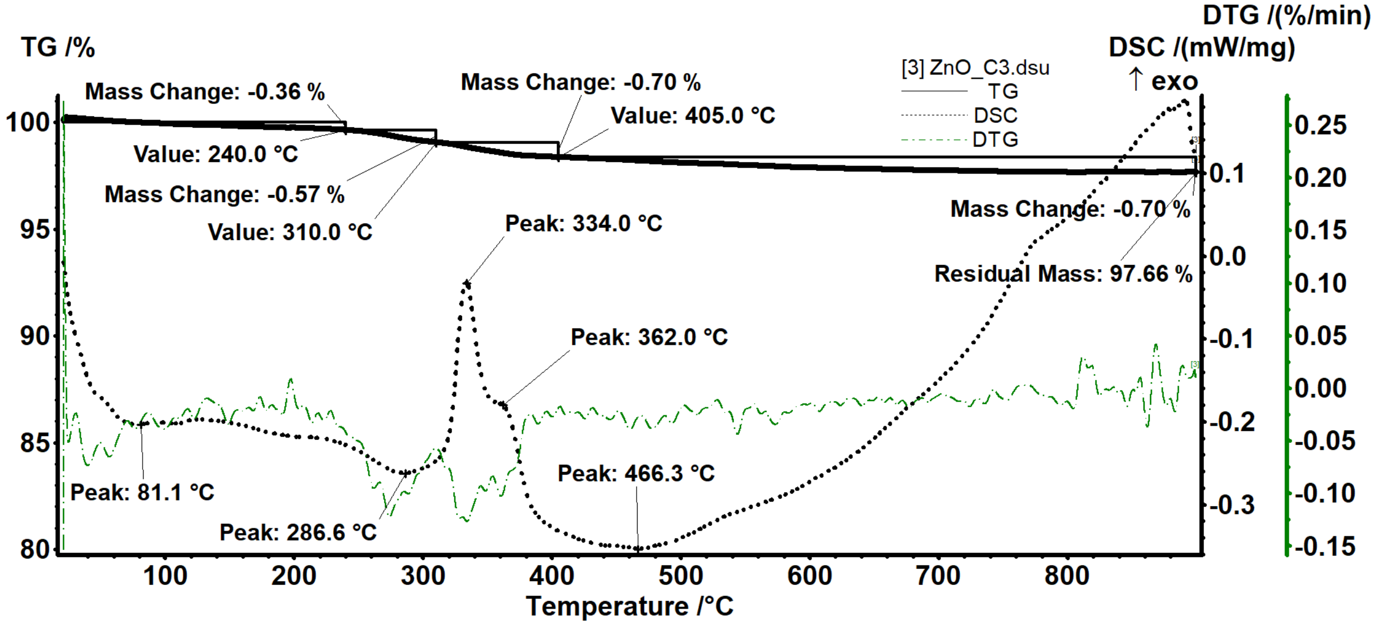

Figure S3. The thermal analysis, TG –DTG – DSC for the sample ZnO_C3 – the nanopowder obtained from zinc acetate by using 1-propanol as solvent.

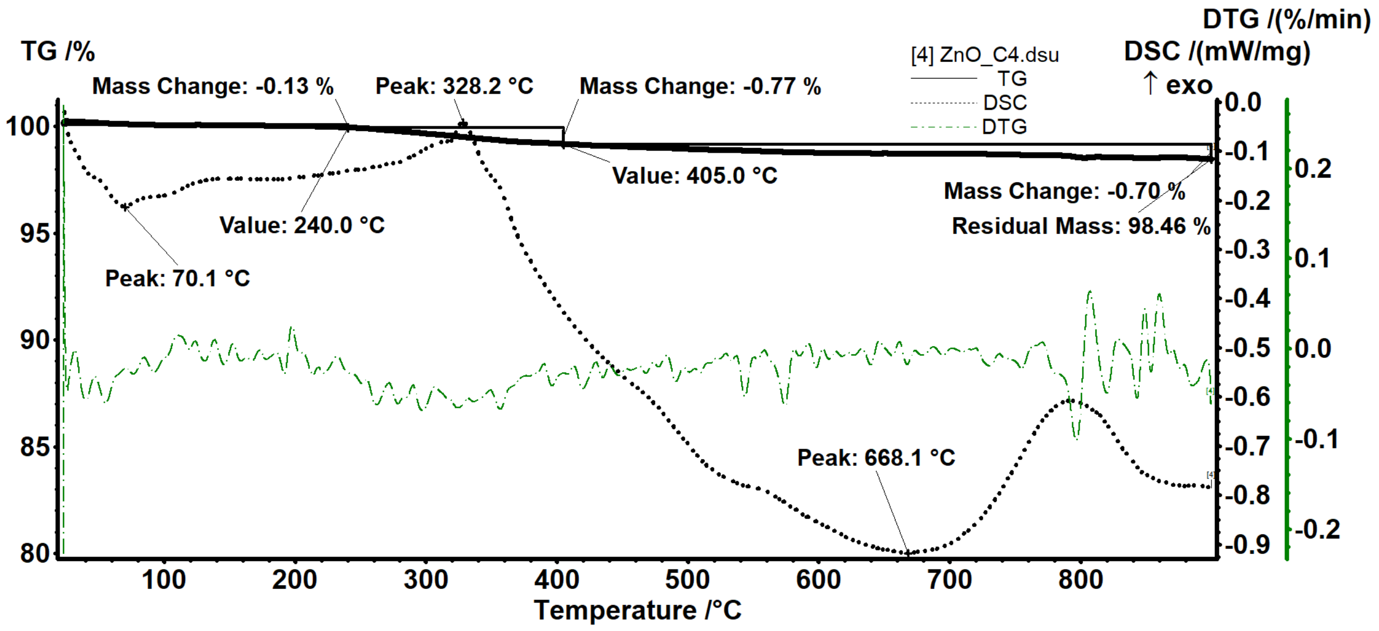

Figure S4. The thermal analysis, TG –DTG – DSC for the sample ZnO_C4 – the nanopowder obtained from zinc acetate by using 1-butanol as solvent.

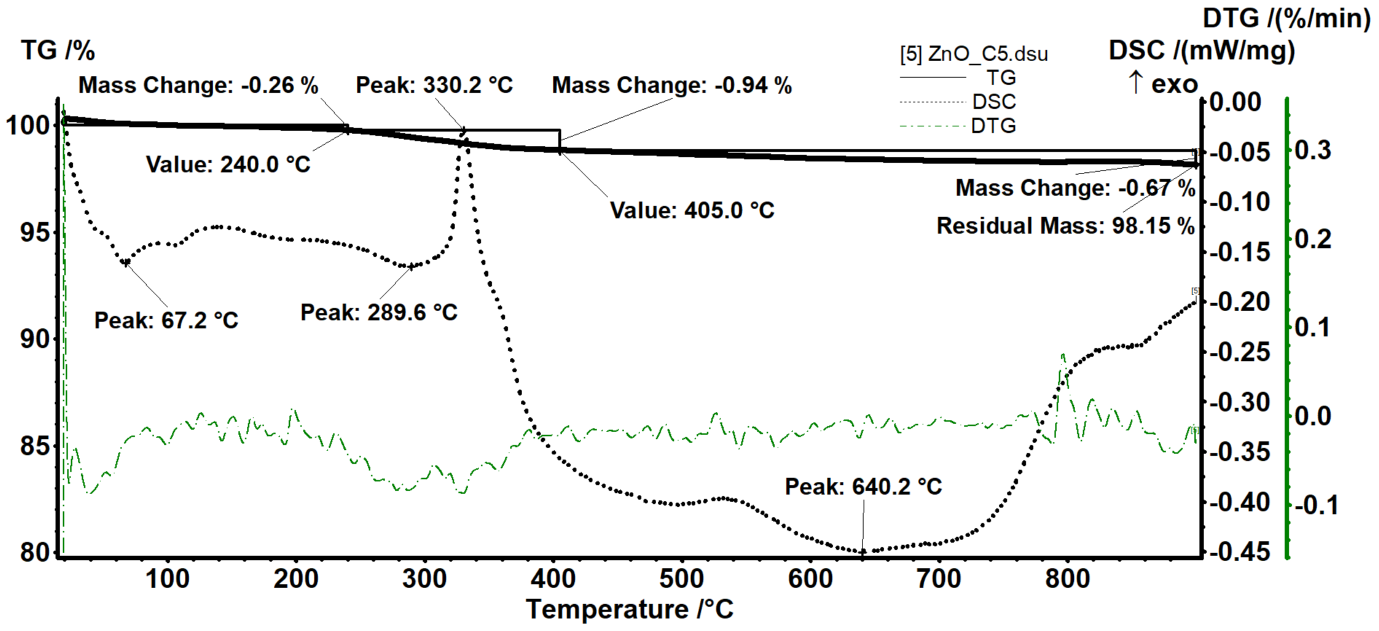

Figure S5. The thermal analysis, TG –DTG – DSC for the sample ZnO_C5 – the nanopowder obtained from zinc acetate by using 1-pentanol as solvent.

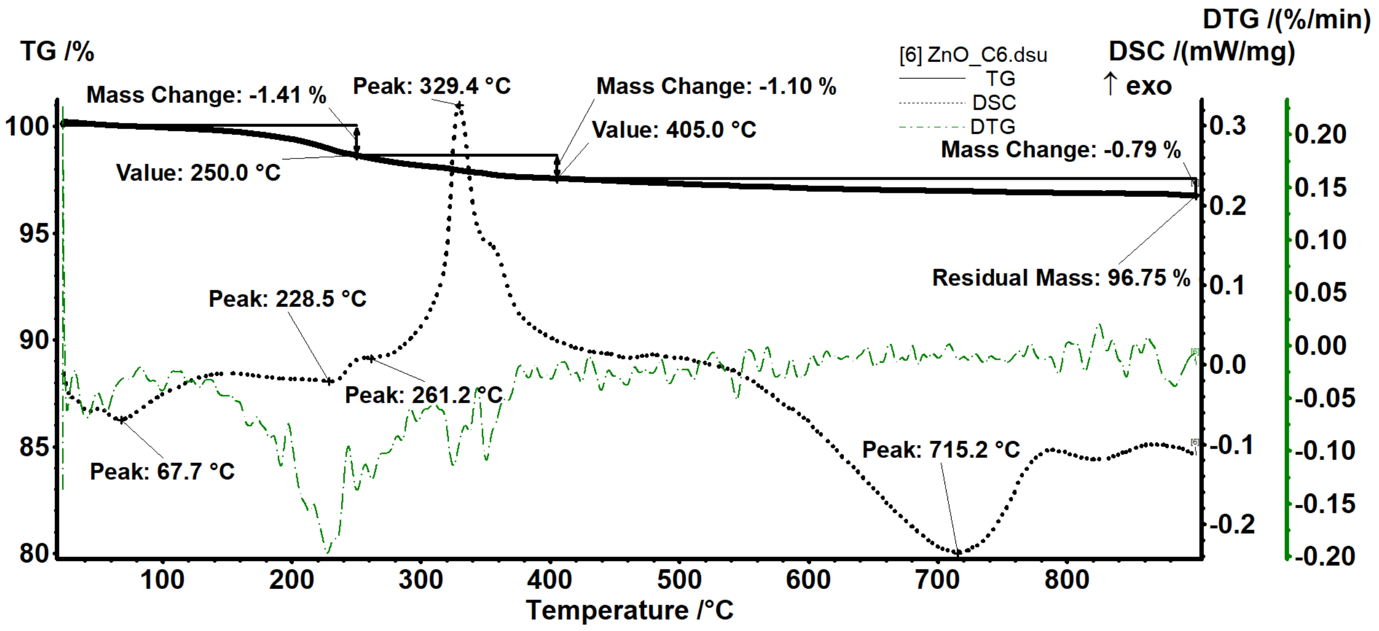

Figure S6. The thermal analysis, TG –DTG – DSC for the sample ZnO_C6 – the nanopowder obtained from zinc acetate by using 1-hexanol as solvent.

Table S1. Principal data obtained from thermal analysis of the nanopowders obtained in primary alcohols (ZnO_C1 in methanol; ZnO_C2 in ethanol; ZnO_C3 in 1-propanol; ZnO_C4 in 1-butanol; ZnO_C5 in 1-pentanol; ZnO_C6 in 1-hexanol).

**Sample label**	Mass loss (20-240°C)	Endo (°C)	Mass loss 240-310°C	Endo (°C)	Mass loss 310-405°C	Exo (°C)	Mass loss (405-900°C)	Endo (°C)
**ZnO_C1**	1.48%	74.2°C	1.58%	281.3°C	1.68%	354.6°C	1.26%	676.5°C
**ZnO_C2**	1.22%	77.3°C	0.85%	281.6°C	0.93%	345.0°C	1.02%	473.4°C
**ZnO_C3**	0.36%	81.1°C	0.57%	286.6°C	0.70%	334.0°C	0.70%	466.3°C
**ZnO_C4**	0.13%	70.1°C	[a]	-	0.77%[a]	328.2°C	0.70%	668.1°C
**ZnO_C5**	0.26%	67.2°C	[a]	289.6°C	0.94%[a]	330.2°C	0.67%	640.2°C
**ZnO_C6**	1.41%[b]	67.7°C	[a,b]	261.2°C	1.10%[a,b]	329.4°C	0.79%	715.2°C

[a]For the samples ZnO_C4, ZnO_C5 and ZnO_C6 the mass loss from 240-310°C is included in the figures from 310-405°C.

[b]For the ZnO_C6 sample the measurement limit for first and second mass loss interval is 250°C.
